# E627V mutation in PB2 protein promotes the mammalian adaptation of novel H10N3 avian influenza virus

**DOI:** 10.1186/s13567-025-01534-8

**Published:** 2025-06-08

**Authors:** Meishan Song, Jianyu Liang, Sige Wang, Ruyi Gao, Xiaolong Lu, Wenhao Yang, Yu Chen, Jingxia Ma, Min Gu, Jiao Hu, Xiaowen Liu, Shunlin Hu, Xiaoquan Wang, Kaituo Liu, Xiufan Liu

**Affiliations:** 1 Joint International Research Laboratory of Agriculture and Agri-Product Safety, The Ministry of Education of China, College of Veterinary Medicine, Yangzhou University, Yangzhou, 225009 China; 2 Key Laboratory of Avian Bioproducts Development, Ministry of Agriculture and Rural Affairs, Yangzhou University, Yangzhou, 225009 China; 3 Jiangsu Co-Innovation Center for Prevention and Control of Important Animal Infectious Diseases and Zoonosis, Yangzhou University, Yangzhou, 225009 China; 4 Jiangsu Key Laboratory of Zoonosis, Yangzhou, 225009 China; 5 Binzhou Argo-Tech Extension Center, Binzhou, 256600 China

**Keywords:** Avian influenza virus, novel H10N3, mammal adaption, PB2-E627V/K

## Abstract

Since 2021, the novel H10N3 has caused four cases of human infection in China, the most recent of which occurred in December 2024, posing a potential threat to public health. Our previous studies indicated that several avian H10N3 strains are highly pathogenic in mice and can be transmitted between mammals via respiratory droplets without prior adaptation. By analyzing the genome sequence, we found that these H10N3 viruses carry the PB2-E627V mutation, which is becoming increasingly common in several subtypes of avian influenza viruses (AIV); however, its mechanism in mammalian adaptation remains unclear. Using a reverse genetics system, we investigated the role of PB2-E627V in the adaptation of H10N3 to mammals and poultry. Our findings demonstrate that the PB2-E627V mutation is critical for the high pathogenicity of novel H10N3 in mice and its ability to be transmitted through the air among mammals. Additionally, we found that the role of PB2-627 V in promoting AIV adaptation to mammals is comparable to that of PB2-627 K. More importantly, PB2-627 V appears to be equally suited to long-term persistence in poultry. Therefore, using PB2-627 V as a novel molecular marker to assess the epidemic potential of AIV is of great significance for preventing possible influenza pandemics in the future.

**Supplementary Information:**

The online version contains supplementary material available at 10.1186/s13567-025-01534-8.

## Introduction

Avian Influenza Viruses (AIV) are primarily hosted by avian species; however, these viruses can undergo extensive cross-species transmission, infecting various mammals, including humans, thereby posing a serious threat to public health security [1]. Multiple subtypes of AIV have been reported to infect humans, with H5Ny and H7N9 attracting significant attention due to the high infection rates and mortality associated with these strains [2–4]. Moreover, in recent years, there have been the first reported cases of human infections caused by subtypes H7N4, H10N3, H3N8, and H10N5 [5–8].

AIV must overcome multiple host restrictions to cross species barriers and infect humans [1, 9]. The initial step for AIV to replicate within a host involves binding to sialic acid receptors on the surface of the host’s respiratory epithelial cells, facilitating viral entry [10–12]. Despite the differences in influenza virus receptor types that are distributed in the respiratory tracts of humans and birds, numerous AIV subtypes currently possess the capability to bind to both α-2,3 and α-2,6 sialic acid receptors [13–15]. This indicates that AIV may have partially overcome the restrictive factor of binding to mammalian cells. Subsequently, upon entering the cells, AIV release viral ribonucleoprotein complexes (vRNP) from the endosome [16]. The vRNA enclosed within these vRNP serve as a template for genomic transcription and replication. However, due to the influence of multiple host restriction factors, the ability of AIV polymerase to replicate its genome in mammalian cells is markedly reduced, preventing high-level replication within these cells. Throughout the process of viral evolution, numerous host-adaptive mutations in viral polymerase proteins have been identified, which mitigate this crucial host barrier to some extent [17, 18]. Several studies have shown that the 627th amino acid residue of the PB2 protein plays a crucial role in the adaptation of AIV to mammalian hosts [19–22].

In recent years, the H10N3 AIV subtype has continued to circulate in China, resulting in four human infections and widespread public concern [13, 23–27]. Our prior research has identified that certain avian-origin H10N3 strains can infect and lethally impact mice without prior adaptation and can be transmitted among guinea pigs via aerosol routes [23]. This indicates that H10N3 possesses the potential for pandemic spread among human populations. Through genomic comparisons, we have discovered the presence of the PB2-627 V molecular marker in these H10N3 strains. While the role of PB2-E627K on the biological characteristics of AIV has been well-documented through extensive research [20, 28–31], the impact of the PB2-E627V has been investigated in only a limited number of studies, with a particular focus on its role in mammalian adaptation, including findings that the PB2-E627V mutation can enhance the replication of H1N1, H9N2, and H5N1 in mammals, and enhance the transmissibility of H7N9 in ferrets [21, 22, 32, 33]. In contrast to previous studies, we found that the PB2-E627V mutation alone is sufficient to enhance H10N3 pathogenicity and transmissibility in mammals, and does not affect virus adaptability in poultry. Moreover, the prevalence of PB2-627 V among avian-origin AIV strains is notably higher than PB2-627 K, thus posing a greater threat to public health security.

## Materials and methods

### Viruses and cells

Avian H10N3 virus A/chicken /Jiangsu/0110/2021 was identified from chickens in Jiangsu [23], and stored at −80 °C after propagation for future use. Madin-Darby canine kidney (MDCK), Human embryonic kidney (HEK293T), and DF1 cells were obtained from the American Type Culture Collection and maintained in Dulbecco modified Eagle medium (Invitrogen, USA) supplemented with 10% fetal bovine serum (Gibco, New Zealand).

### Generation of mutant viruses by reverse genetics

All gene segments of A/chicken/Jiangsu/0110/2019 were cloned into the pHW2000 vector, as previously described [34]. Mutations in the PB2 protein (V627K and V627E) of the 0110-WT, were generated using the Fast Mutagenesis System kit (TransGene, China). Eight plasmids were co-transfected into HEK293T cells using the Lipofectamine 2000 Reagent (Invitrogen, USA). Culture supernatant aliquots were harvested at 72 h post-transfection, and 9-day-old chicken embryos were infected and incubated for 96 h. Parental and mutant viruses were purified for three rounds and confirmed by sequencing. Aliquots of viral fluid were stored at −80 ℃.

### Mouse challenge experiments

Six-week-old female BALB/c mice (Yangzhou Experimental Animal Center, Jiangsu, China) were used as animal models to investigate the pathogenicity of the indicated viruses to mammals. Groups of five mice were anesthetized with Zoletil50 (tiletamine-zolazepam [Virbac, Carros, France], 20 mg/g body weight) and intranasally (*i.n.*) inoculated with 50 µL virus in tenfold serial dilutions from 10^1.0^ to 10^6.0^ EID_50_. All mice were monitored daily for 14 days and for mortality to determine the mouse lethal dose at 50% (MLD_50_). Mice that lost ≥ 25% of their original body weight were humanely euthanized. The MLD_50_ values were calculated according to the method of Reed and Muench [35]. To monitor virus production, three mice infected with 10^5.0^ EID_50_ from each group were euthanized at 3 days post-infection (dpi), respectively. Lung edema was assessed using the lung wet/dry weight ratio. Briefly, the lungs of each virus-inoculated or control mouse were collected and weighed to obtain the wet mass. The lungs were heated to 65 °C in a gravity convection oven for 24 h and weighed to obtain the dry mass. The wet/dry weight ratio was calculated by dividing the dry mass by the wet mass. Nasal turbinate, lung, brain, and kidney samples were collected for virus titration. Lungs of mice infected with the indicated viruses at 3 dpi were collected, and fixed in 10% neutral-buffered formalin, embedded in paraffin, and cut into 4 µm sections. The sections were stained with hematoxylin–eosin (H&E) assays.

### Guinea pig transmission experiments

SPF Hartley-strain female guinea pigs, weighing 250–300 g (Vital River Laboratories, Beijing, China) were used to investigate the transmissibility of these viruses in mammals. Groups of three guinea pigs were anesthetized and inoculated *i.n*. with 10^5.0^ EID_50_ of the desired viruses in a volume of 200 μL. At 24 hpi, three additional naive guinea pigs were placed into the same isolation units for the direct-contact transmission studies, and another three naive guinea pigs per group were housed in adjacent cages (5 cm apart) to monitor airborne transmission [13]. The airflow rates from the infected group to the exposed group were adjusted to > 0.1 to approximately < 0.3 m/s. The temperature and humidity were set at 22 °C and 30 to 40%. To monitor virus shedding, nasal washes were collected from all guinea pigs every other day for 12 days and titrated for viruses on 9-day-old embryonated SPF chicken eggs. Sera were collected from all guinea pigs at 21 dpi and treated with receptor-destroying enzyme (DENKA SEIKEN, Japan); seroconversion was analyzed by HI assay.

### Chicken challenge experiments

Groups of six four-week-old SPF chickens were inoculated *i.n.* with 10^6.0^ EID_50_ of the desired viruses in a volume of 100 μL to investigate the transmissibility of the viruses in chickens. At 24 h post-infection (hpi), three additional naive chickens were placed into the same isolation units for the direct-contact transmission studies, and another three naive chickens per group were housed in adjacent cages (5 cm apart) to monitor airborne transmission. To monitor virus shedding, oropharyngeal and cloacal swabs were collected from all chickens every other day for 10 days. Sera were collected from both inoculated and contacted chickens at 14 dpi. Seroconversion was analyzed by hemagglutination inhibition (HI) assay. Additionally, three chickens in the inoculated group were euthanized, and the trachea, liver, lungs and intestines were collected aseptically at 5 dpi for viral titration. Nine-day-old embryonated SPF chicken eggs were used for virus titration.

### Polymerase activity assay

Viral polymerase activity was determined using a luciferase reporting assay. Ribonucleoprotein (RNP) complexes composed of PB2, PB1, PA and NP derived from A/chicken/Jiangsu/0110/2019 and 0110 mutants were separately cloned into the pcDNA3.1(+) vector. Cells were co-transfected with PB2, PB1, NP, and PA expression plasmids (200 ng) together with the p-Luci plasmid (p-Luci, carrying an IAV reporter minigenome in which the firefly luciferase gene is flanked by the non-coding regions of the NS gene from IAV, a truncated PolI promoter and the hepatitis delta virus ribozyme, described previously [36], 300 ng) or paviPolIT-Luc (paviPolIT-Luc, containing the firefly luciferase gene flanked by the non-coding regions of the NP gene of IAV is inserted into a pPolI plasmid housing the 250-nucleotide sequence of the avian polymerase I promoter, described previously [36], 300 ng), and internal control *Renilla* plasmid (pRL-TK, 30 ng). As a negative control, the PB1, NP, and PA expression plasmids were transfected into desired cells together with the p-Luci or paviPolIT-Luc plasmid, and internal control *Renilla* plasmid, respectively. At 24 h, cell lysates were processed to measure firefly and *Renilla* luciferase activities using GluMax 96 microplate luminometer (Promega). PB2 proteins were detected by western blotting. Three independent experiments were performed.

### Statistical analysis

Graphing and statistical analyses were performed using GraphPad Prism 9 (GraphPad Software, San Diego, California, USA). One-way ANOVA test was used for one-way comparisons between multiple groups, and two-way ANOVA test was used for two-way comparisons between multiple groups. Statistical significance was reported at *P* < 0.05, *P < 0.05, **P < 0.01, ***P < 0.001, ****P < 0.0001.

## Results

### PB2-627 V/K is a critical determinant of novel H10N3 virulence in mammals

**Figure 1:**
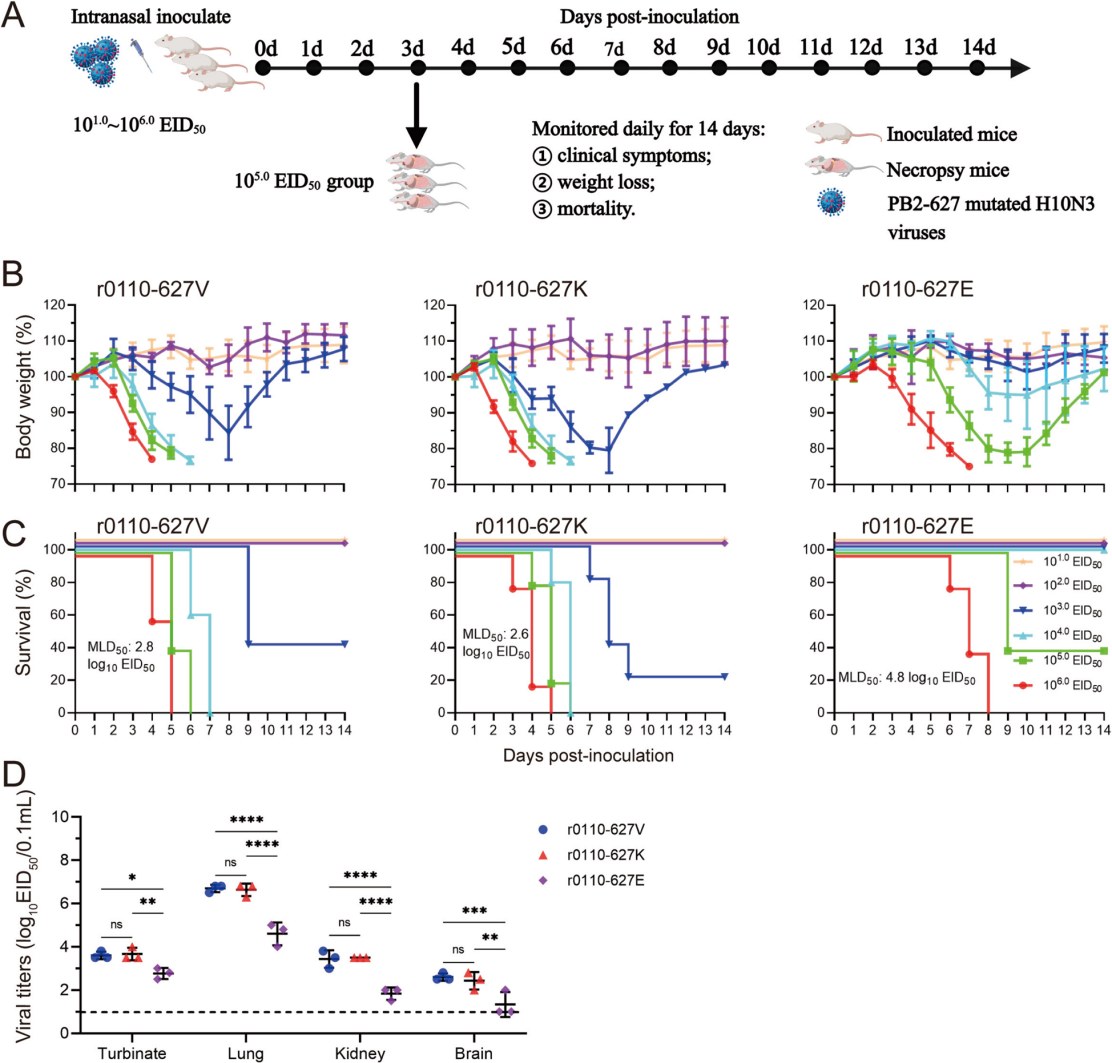
**Virulence and replication of the novel H10N3 viruses with PB2-V/K/E mutation in mice.** **A** Diagram of the experimental procedure. Briefly, groups of six-week-old five BALB/c mice were inoculated *i.n.* with the indicated viruses at doses 10^1.0^ ~ 10^6.0^ EID_50_; Clinical symptoms, body weight and survival were monitored daily for 14 dpi; mice were humanly killed when they lost ≥ 25% of their initial body weight; 10^5.0^ EID_50_ group mice were dissected and sampled on day 3 to detect the viral loading. **B** Body weight loss was monitored for 14 days. **C** Survival was monitored for 14 days. **D** Mice were challenged with 10^5.0^ EID_50_ of the indicated virus, and three mice per group were euthanized at 3 dpi; Virus titers in different organs (turbinate, lung, kidney and brain) were determined by EID_50_ assay on chicken eggs; Horizontal dashed lines indicate the lower limit of detection. Data are represented as mean ± SD.

Pathogenicity and transmission of influenza virus in mammals is a complex polygenic and polyfactorial process [37, 38], the 627^th^ position of the PB2 protein is one of the key sites that has been proven to play a significant role [19, 20]. In our previous study, chicken-derived H10N3 isolate, CK/0110/19, exhibited a PB2-E627V substitution, showing high pathogenicity in mice and effective transmission through both direct contact and respiratory droplets [23]. To investigate the role of the 627th amino acid in the PB2 protein in the pathogenicity and transmission of novel H10N3, we constructed the r0110-627E and r0110-627 K mutant viruses using the 0110 strain (r0110-627 V) as the parent. As shown in Figures 1B and C, pathogenicity experiments in mice revealed that the r0110-627 K variant (MLD_50_ = 2.6 log_10_ EID_50_) exhibits a similar level of pathogenicity as r0110-627 V (MLD_50_ = 2.8 log_10_ EID_50_), whereas the r0110-627E variant displays a significantly lower level of pathogenicity, with a reduction factor of 100 yielding an MLD_50_ = 4.8 log_10_ EID_50_. This decrease in pathogenicity is corroborated by the markedly lower replication levels of the r0110-627E strain in mouse organs, including turbinate, lung, kidney and brain (Figure 1D). Overall, while PB2-627 V/K plays a crucial role in the high pathogenicity of novel H10N3 strains in mice, it is not the sole determinant.

### PB2-627 V/K causes more severe lung injury and inflammatory cytokine storm in mice

**Figure 2:**
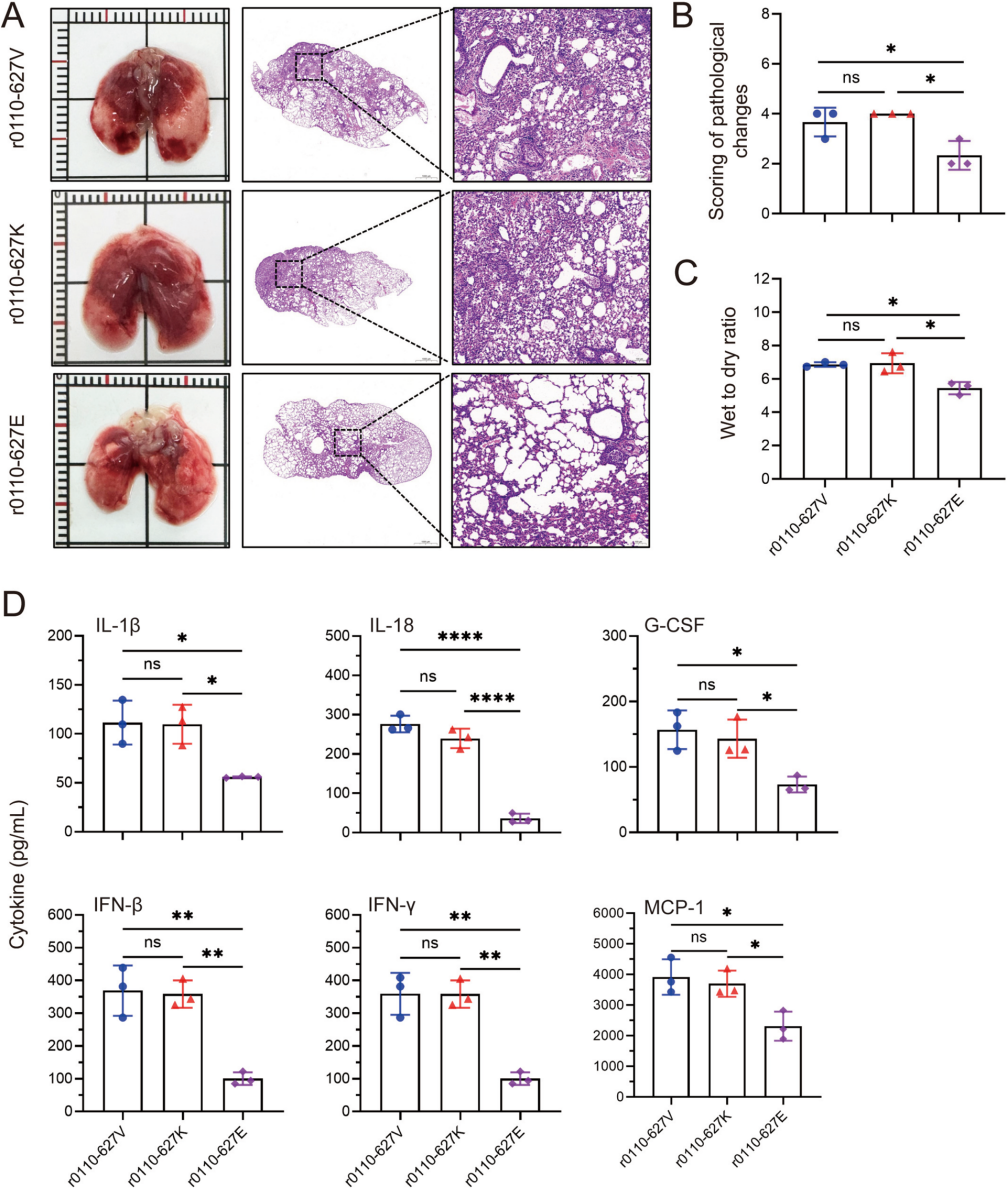
**Lung lesions and inflammatory cytokine of the novel H10N3 viruses with PB2-V/K/E mutation in mice.** **A** Macroscopic lesions and histopathological (lung sections were stained with H&E) findings in the lungs of mice infected with the indicated viruses at an EID_50_ of 10^5.0^ at 3 dpi. **B** Histopathological scores in the lungs of mice inoculated with indicated viruses at doses 10^5.0^ EID_50_ at 3 dpi. Lung tissue sections were scored based on pathological changes: 0, no visible lesions; 1, affected area by the lesions (< 10%); 2, affected area by the lesions (< 30%, ≥ 10%); 3, affected area by the lesions (< 50%, ≥ 30%); 4, affected area by the lesions (≥ 50%). **C** Lung edema was assessed using lung wet/dry weight ratio. **D** Lung lavage samples from three mice in each virus-infected group (challenged with 10^5.0^ EID_50_) were collected for analysis of Inflammatory cytokine on 3 dpi. Data are represented as mean ± SD.

Influenza virus infections in mammalian hosts primarily affect the respiratory system, with the lungs being a principal target organ. Such infections can precipitate pneumonia or acute respiratory distress syndrome (ARDS), leading to respiratory complications and potentially mortality. In terms of macroscopic pathology, the PB2-627 V/K/E mutant viruses consistently produce dark red pulmonary consolidation (Figure 2A). Notably, the r0110-627 V and r0110-627 K mutations result in lesions affecting more than 50% of the lung parenchyma, whereas the r0110-627E mutation causes lesions in approximately 30% of the lung tissue (Figures 2A and B). Histopathological analysis of lung specimens has demonstrated that all three viral mutations induce significant pathological changes, including thickening of alveolar septa, capillary hemorrhage, and infiltration of inflammatory cells (Figure 2A). However, the extent of these histopathological abnormalities was markedly greater in infections with the r0110-627 V and r0110-627 K mutations compared to r0110-627E. Furthermore, assessments of pulmonary edema through dry-to-wet weight ratio analysis revealed that the r0110-627 V and r0110-627 K strains are associated with more severe pulmonary edema (Figure 2C). Pulmonary edema is usually caused by an excessive inflammatory response, so we measured the expression of inflammatory factors in bronchoalveolar lavage fluid. The levels of IL-1β, IL-18, G-CSF, IFN-β, IFN-γ and MCP-1, in the lungs of r0110-627 V and r0110-627 K-inoculated group were all significantly higher than those in the lungs of the r0110-627E-inoculated groups (Figure 2D). Moreover, the levels of only two cytokines (IL-6 and TNF-α) in the lungs of the r0110-627 V-inoculated group were significantly higher than those in the r0110-627 K-inoculated group. In addition they have four cytokines and chemokines (CXCL1, IL-10, IFN-α and MIP-α) and show no significant differences in the lungs between the three mutated viruses inoculated group (Additional file 1). These results indicate that PB2-627 V/K could enhance H10N3 AIV subtype causing more severe lung injury and an inflammatory cytokine storm in mammals.

### PB2-627 V/K is a critical determinant of novel H10N3 transmission in mammals

**Fig. 3:**
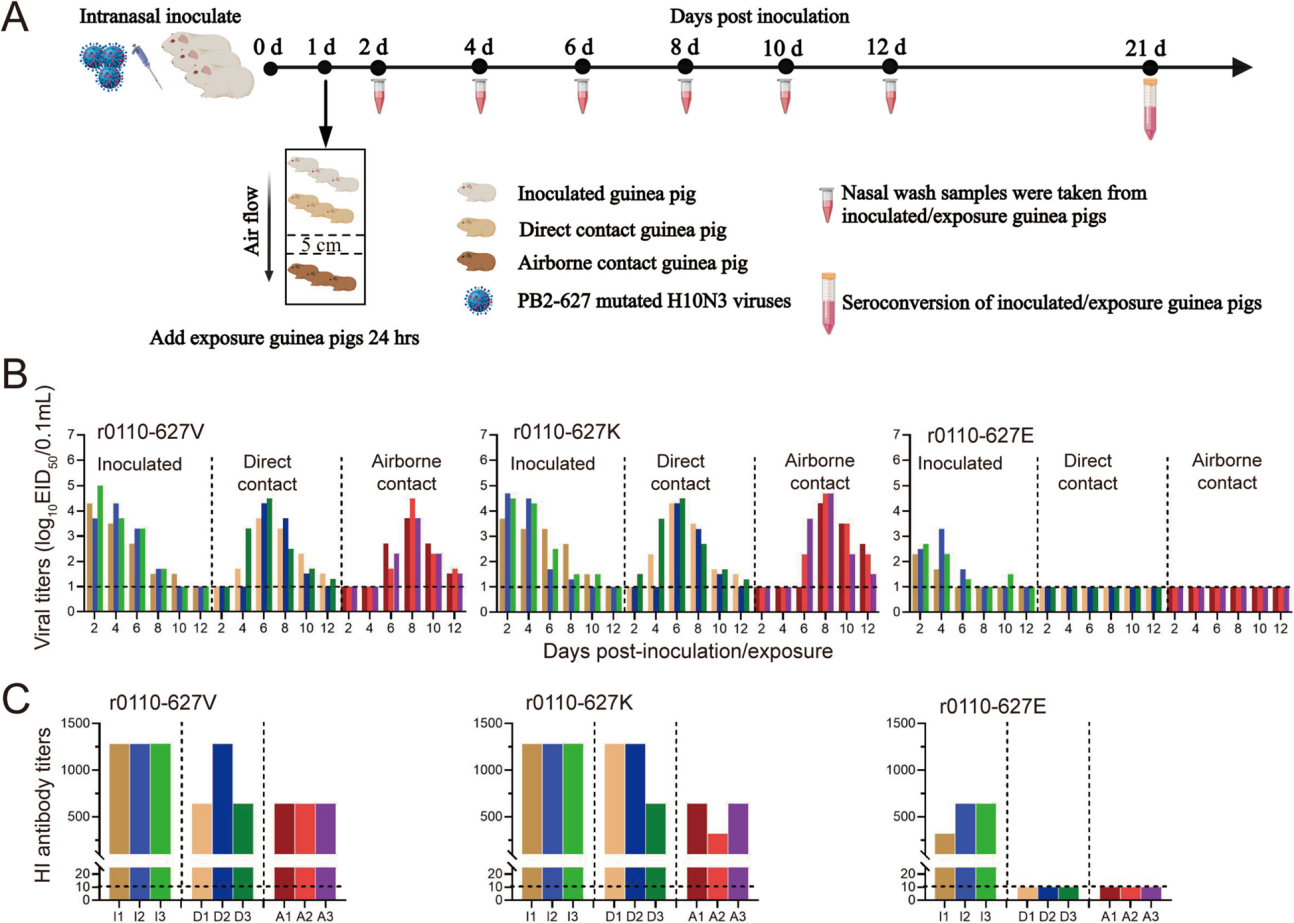
**Horizontal transmission of the novel H10N3 viruses with PB2-V/K/E mutation in guinea pigs.** **A** Diagram of the experimental procedure. Briefly, Groups of three guinea pigs were anesthetized and inoculated *i.n.* with 10^5.0^ EID_50_ of the indicated viruses, at 24 hpi, three additional guinea pigs were placed into the same isolation units for the direct-contact transmission studies, and another three guinea pigs were housed in adjacent cages (5 cm apart) to monitor airborne transmission; Nasal washes for virus shedding detection were collected every other day from all animals from 2 dpi after initial infection and titrated in chicken eggs; Seroconversion was analyzed by HI assay in 21 dpi. **B** Virus shedding of infected and exposed guinea pigs. Virus titers were determined by EID_50_ assay in chicken eggs; Each color bar represents the virus titer in an individual animal. **C** Seroconversion of infected and exposed guinea pigs. Serum was treated with receptor-destroying enzyme and analyzed by HI assay; I, infected; D, direct-contact; A, airborne-contact. Horizontal dashed lines indicate the lower limit of detection.

We also examined the impact of the PB2-627 V/K/E mutation on the transmission of the novel H10N3 virus in guinea pigs. As shown in Figure 3, transmission experiments revealed that the PB2-V627K mutation does not affect the transmissibility of the novel H10N3 virus in guinea pigs. However, r0110-627E lost its ability to transmit amongst guinea pigs, including both direct- and airborne-contact (Figure 3), suggesting that the V627E mutation in PB2 can confer transmission of novel H10N3 viruses. In addition, the virus shedding titers from nasal of the r0110-627 V and r0110-627 K-inoculated groups were all significantly higher than the r0110-627E-inoculated groups (Figure 3B). Serological analysis also indicates that r0110-627 V and r0110-627 K could induce higher antibody titers in guinea pigs (Figure 3C). In summary, PB2-627 V/K was found to contribute to the transmission of novel H10N3 viruses between mammals.

### PB2-627 V/K/E does not affect the transmissibility of novel H10N3 virus in chickens

**Figure 4:**
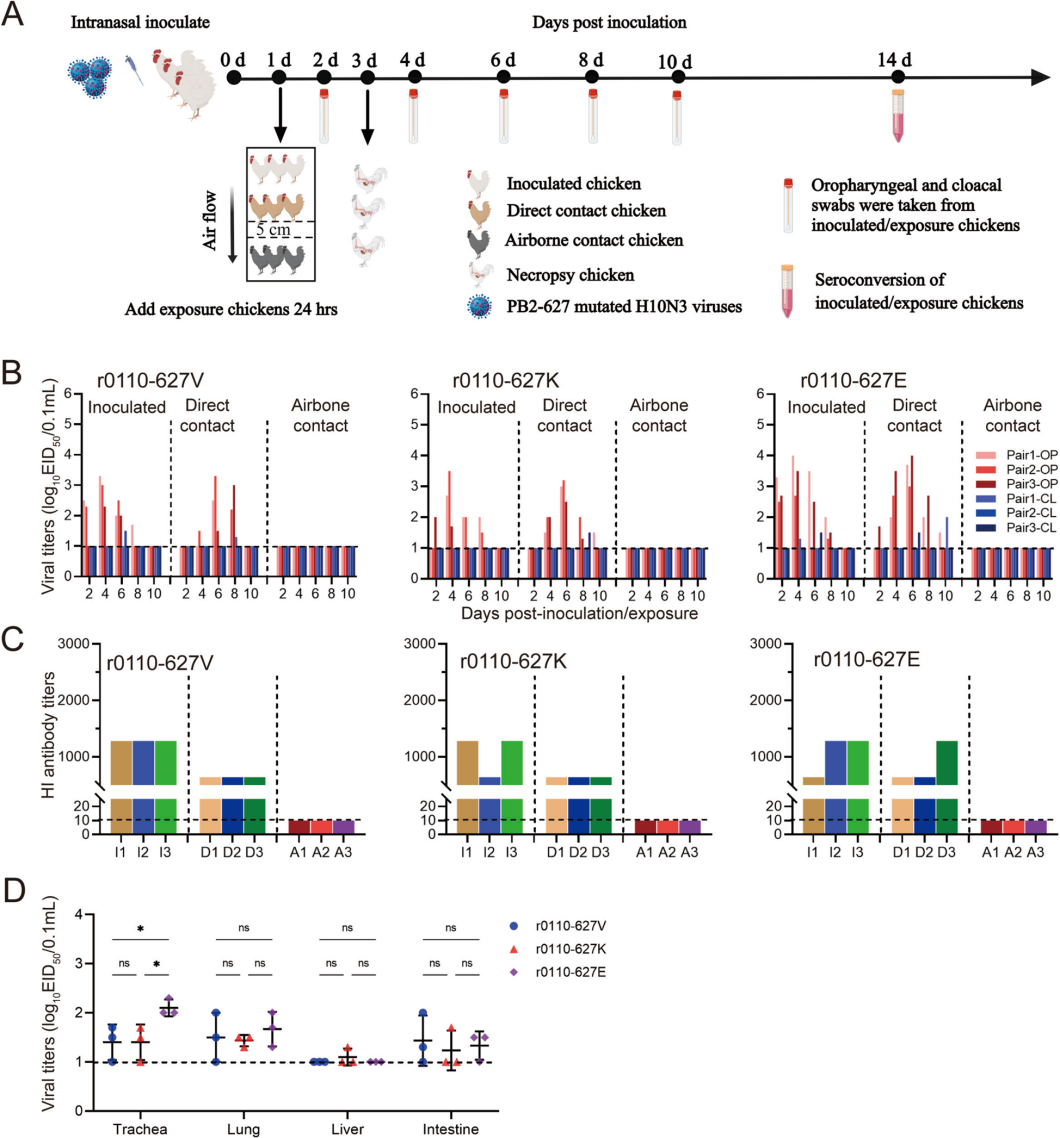
**Horizontal transmission and replication of the novel H10N3 viruses with PB2-V/K/E mutation in chickens.** **A** Diagram of the experimental procedure. Briefly, Groups of three chickens were inoculated *i.n.* with 10^6.0^ EID_50_ of the indicated viruses, at 24 hpi, three additional chickens were placed into the same isolation units for the direct-contact transmission studies, and another three chickens were housed in adjacent cages (5 cm apart) to monitor airborne transmission; Oropharyngeal (OP) and cloacal (CL) swabs for virus shedding detection were collected every other day from all chickens from 2 days after initial infection; Seroconversion was analyzed by HI assay in 14 dpi. **B** Virus shedding of infected and exposed chickens. Virus titers were determined by EID_50_ assay in chicken eggs; Each color bar represents the virus titer from oropharyngeal and cloacal swabs from an individual animal. **C** Seroconversion of infected and exposed chickens. I, infected; C, contacted; A, airborne-contacted. Horizontal dashed lines indicate the lower limit of detection. **D** Chickens were challenged with 10^6.0^ EID_50_ of indicated virus, and three chickens per group were euthanized at 5 dpi; Virus titers in different organs (trachea, lung, liver and intestine) were determined by EID_50_ assay on chicken eggs; Horizontal dashed lines indicate the lower limit of detection. Data are represented as mean ± SD.

The widespread prevalence of AIV in chickens is a significant factor contributing to human infections. Thus, the effect of PB2-627 on replication and transmission in chickens was studied. To evaluate the effect of PB2-627 on the replication of AIV in vivo, the chickens were infected with 10^6.0^ EID_50_ viruses, and trachea, lungs, livers and intestines were collected for virus titration at 5 dpi. As shown in Figure 4D, the replication levels of r0110-627E in the trachea were significantly higher than that of strains r0110-627 V and r0110-627 K, while there was no difference in replication levels in the other organs tested. Virus transmission was determined by titration of nasal washes in eggs and testing for seroconversion at 14 dpi. As shown in Figure 4B, three mutated virus-infected chickens all shed the virus through the throat and cloaca; virus shedding began at 2 dpi and persisted for 8 days. However, the peak titer of r0110-627E was 10^4.0^ EID_50_/0.1 mL, while those of r0110-627 V and r0110-627 K were only 10^3.3^ EID_50_/0.1 mL and 10^3.5^ EID_50_/0.1 mL, respectively. All the direct-contact chickens exhibited viruses shedding and had seroconverted by 14 dpi, and no virus could be detected in any airborne-contact chickens (Figure 4D), indicating that the PB2-627 V/E/K mutation does not affect the transmissibility of novel H10N3 virus in chickens.

### PB2-627 V/K/E mutations regulate the replication of novel H10N3 in different cell lines

**Figure 5:**
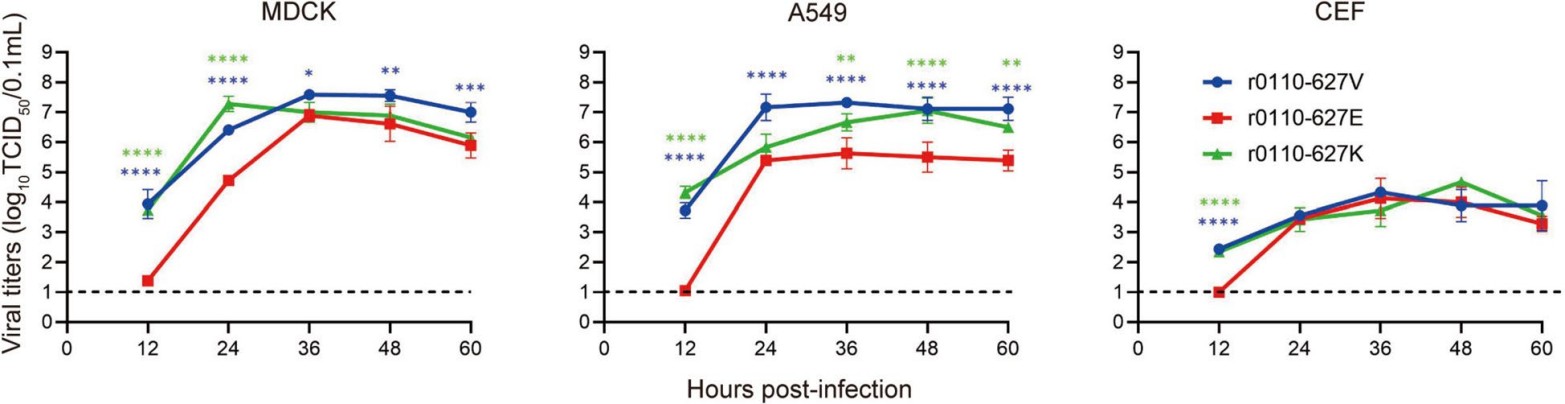
**Growth dynamics of the novel H10N3 viruses with PB2-V/K/E mutation in different cell lines.** MDCK, A549, and CEF were inoculated with the indicated viruses at a multiplicity of infection (MOI) of 0.01 TCID_50_/cell in triplicate at 37 °C. Supernatants were aseptically harvested at 12, 24, 36, 48 and 60 hpi. The virus titers were determined by performing TCID_50_ per 0.1 mL in MDCK cells. Horizontal dashed lines indicate the lower limit of detection. Data are represented as mean ± SD.

To evaluate the effect of PB2-627 on the replication of AIV in vitro, replication ability of the PB2-627 mutated viruses were tested in different host cell lines. Mammalian cells (MDCK and A549) and avian cells (CEF) were inoculated with the indicated viruses at an MOI of 0.01, to quantify the viral replication dynamics during an overt infection (8–60 hpi). As shown in Figure 5, in MDCK and A549 cells, H10N3 viruses carrying the PB2-627 V/K mutation (r0110-627 V and r0110-627 K) generated significantly higher titers than the r0110-627E virus. Additionally, r0110-627 V and r0110-627 K also generated significantly higher titers than the r0110-627E virus during the initial infection stages (12 hpi), whereas the viral titers did not significantly differ during the middle and late infection stages. These results indicate that the PB2-627 V/K mutation increased H10N3 AIV subtype replication in different host cell lines.

### PB2-627 V/K/E mutations regulate the polymerase complex activity of novel H10N3 virus

**Figure 6:**
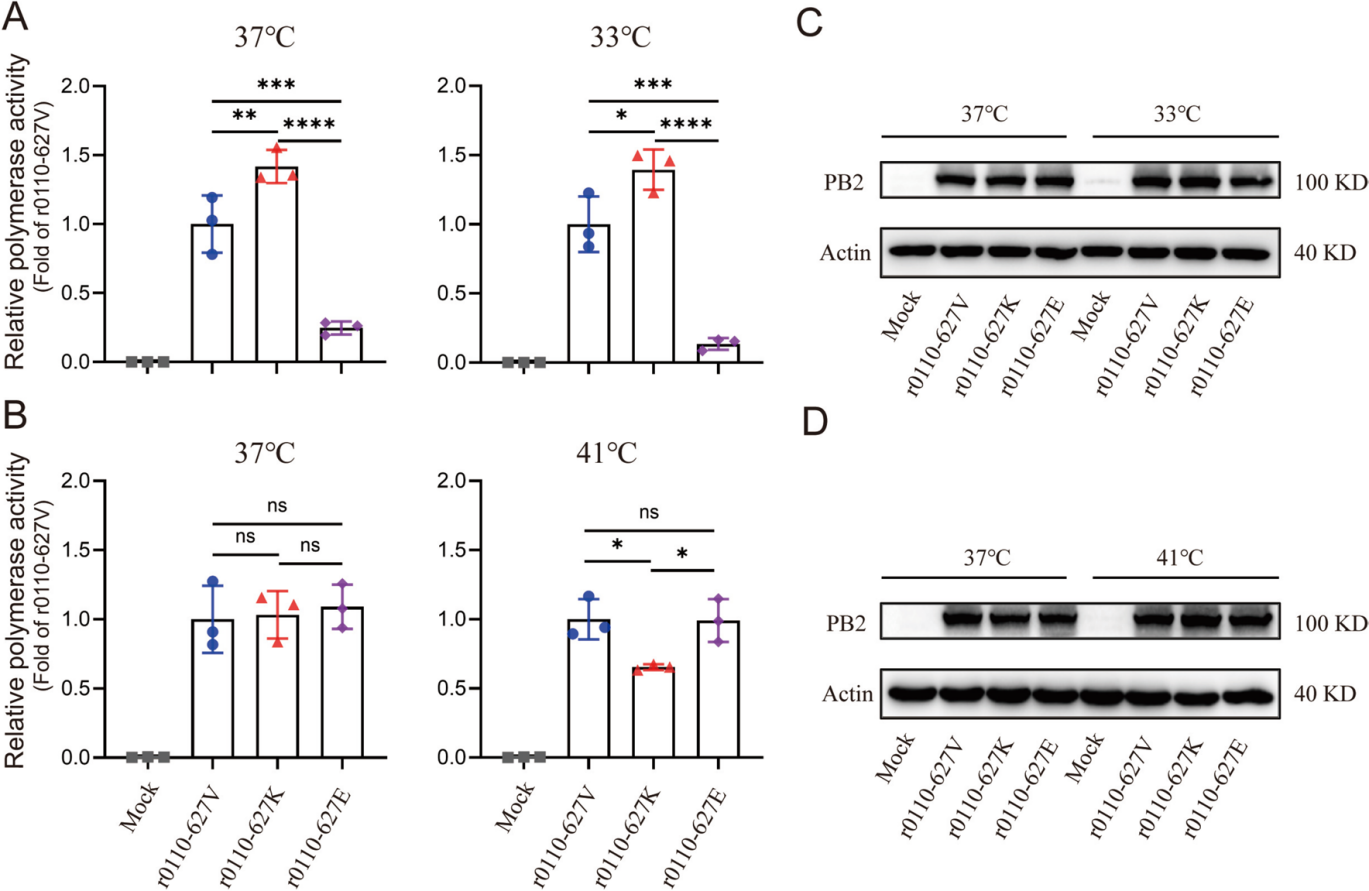
**Effect of indicated PB2-627 V/K/E mutations on polymerase complex activity.** Plasmids encoding PB2, PB1, PA, and NP proteins derived from A/chicken/Jiangsu/0110/2019 (0110) with the indicated amino acid substitutions (PB2- 627 V/K/E) were transfected into cells at the indicated temperature, together with the P-Luci or paviPolIT-Luc plasmid and internal control plasmid (pRL-TK). As negative control, the MOCK group was transfected with the PB1, NP, and PA expression plasmids, the p-Luci or paviPolI-Luc plasmid, and internal control Renilla plasmid. At 24 h post-transfection, luciferase activities were measured. **A** Plasmids were transfected into HEK293T cells at 37 ℃ (left) and 33 ℃ (right). **B** Plasmids were transfected into DF1 cells at 37 ℃ (left) and 41 ℃ (right). **C** Levels of PB2 protein in HEK293T were detected by western blotting. **D** Levels of PB2 protein in DF1 was detected by western blotting. Data are represented as mean ± SD.

To ascertain the relative contribution of PB2-627E/V/K to RNP polymerase activity, minigenome replication assays were conducted in HEK293T cells at 33 ℃ and 37 ℃ (corresponding to the temperatures of the human upper and lower respiratory tracts, respectively). As shown in Figure 6A, polymerase activity for r0110-627 K was observed to be 1.39- and 1.42-fold higher than that of r0110-627 V at 33 ℃ and 37 ℃, respectively. Significantly, the polymerase activity of the r0110-627E mutant was 7.42- and 4.05-fold lower than that of PB2-627 V at the same respective temperatures. Additionally, no significant difference was observed in the expression of PB2 proteins after PB2-627 V/K/E mutation (Figure 6C). These findings suggest that the polymerase activities of novel H10N3 viruses possessing PB2-627 V/K amino acid are greater than PB2-627E in mammalian cell lines.

The above result shows that the mutation in PB2-627 also affects the replication of AIV in avian cells. To ascertain the relative contribution of PB2-627 V/K/E to RNP polymerase activity in DF1 cells at 37 °C and 41 ℃ (corresponding to the temperatures of the chicken upper and lower respiratory tracts, respectively), minigenome replication assays were also performed. As shown in Figure 6B, no significant differences in polymerase activity were observed among the r0110-627 V/K/E mutants at 37 ℃. Furthermore, there was no substantial difference in polymerase activities between r0110-627 V and r0110-627E at 41 ℃, however, both displayed higher activities than r0110-627 K (Figure 6B). Additionally, no significant difference was observed in the expression of PB2 proteins after PB2-627 V/K/E mutation (Figure 6D). In conclusion, the polymerase activities of the novel H10N3 viruses possessing the PB2-627 V/E amino acid are higher than those with PB2-627 K in avian cell lines.

### Prevalence of PB2-627 V/K/E in different AIV subtype

We downloaded the PB2 gene sequences of several AIV subtypes from the Global Initiative of Sharing All Influenza Data (GISAID) database, such as H10N3, H5N1, H5N6, H7N9, H9N2, and H3N8, and conducted a systematic analysis of the distribution characteristics of the 627^th^ amino acid across different hosts. As shown in Table 1, in avian-derived strains of all AIV subtypes, PB2-627E is the predominant amino acid, accounting for more than 90% of cases, while the proportions of PB2-627 V/K are generally at lower levels. Notably, the proportion of PB2-627 V is relatively higher in avian-derived H9N2 (6.28%) compared to other AIV subtypes of, and the proportion of PB2-627 K is also higher in avian-derived H5N1 (6.61%) than in other AIV subtypes. In comparison to avian-derived AIV, human-derived strains show a significant decrease in the proportion of PB2-627E and a substantial increase in the proportion of PB2-627 K. In most subtypes, the proportion of PB2-627 K exceeds 30%, and importantly, the proportion of PB2-627 K exceeds 60% in H7N9 human-derived viruses. Interestingly, the proportion of PB2-627 K in human-derived H9N2 strains is relatively low (2.86%), while the level of PB2-627 V is relatively high (31.43%). In conclusion, the notable characteristic of AIV adaptation to mammals is the mutation of the PB2-627 amino acid from E to V.

**Table 1:** **Prevalence of amino acid type at positions 627 of PB2 protein in different AIV subtypes.**

Acid type	Strains^a^(%)											
	H5N1		H5N6		H7N9		H9N2		H10N3^b^		H3N8	
	Avian	Human	Avian	Human	Avian	Human	Avian	Human	Avian	Human	Avian	Human
E	9462 (93.27%)	298 (62.87%)	1924 (97.62%)	24 (54.55%)	983 (98.79%)	350 (25.62%)	5135 (92.89%)	45 (64.29%)	168 (96.55%)	3 (100%)	2043 (99.90%)	1 (33.33%)
V	1 (0.01%)	0 (0.00%)	33 (1.67%)	1 (2.27%)	3 (0.30%)	33 (2.42%)	347 (6.28%)	22 (31.43%)	6 (3.45%)	0 (0.00%)	1 (0.05%)	1 (33.33%)
K	671 (6.61%)	175 (36.92%)	4 (0.20%)	18 (40.91%)	9 (0.90%)	924 (67.64%)	30 (0.54%)	2 (2.86%)	0 (0.00%)	0 (0.00%)	1 (0.05%)	1 (33.33%)
Others	11 (0.11%)	1 (0.21%)	10 (0.51%)	1 (2.27%)	0 (0.00%)	59 (4.32%)	16 (0.29%)	1 (1.43%)	0 (0.00%)	0 (0.00%)	0 (0.00%)	0 (0.00%)
Total	10,145	474	1971	44	995	1366	5528	70	174	3	2045	3

^a^These PB2 protein sequences were download from the Global Initiative of Sharing All Influenza Data (GISAID) database. Duplicate, gapped, or truncated sequences were excluded prior to analysis.

^b^As of January 27, 2025, four cases of human infection with H10N3 have been reported globally. The most recent case occurred on December 12, 2024, in the Guangxi Zhuang Autonomous Region, China. However, the sequence of the Guangxi strain has not yet been uploaded to either the NCBI or GISAID databases and is therefore not included in our analysis.

## Discussion

Many viruses, particularly RNA viruses, can quickly break through host barriers due to high-frequency gene mutations and cause cross-species infections. Understanding how AIV adapt to mammals is crucial for monitoring and preventing future pandemics in humans. The adaptation of AIV to mammalian hosts primarily results from the synergistic effects of multiple genes [37–39]; however, a few sites can independently contribute to this adaptation, with the most prominent being position 627 in the PB2 subunit [19, 20].

The PB2 protein is essential for efficient AIV replication in host cells [40]. Functionally, although the 627th amino acid of the PB2 protein does not participate in the core catalytic activities of the polymerase, it is crucial for recruiting the polymerase to the vRNP and for the binding and stabilization of cRNA by the polymerase [41, 42]. In addition, many host factors, especially mammalian ANP32 proteins, are considered important host factors that restrict the replication of AIV in humans [43, 44]. Compared with PB2-627E, the presence of a basic amino acid (K) at the PB2-627 site enhances the interaction between ANP32 proteins and the PB2-627 domain, and this interaction is essential for supporting polymerase activity [45]. While AIV usually contain PB2-627E, PB2-E627K is a well-recognized mammalian adaptation substitution at this position [28, 33, 46–48]. PB2-E627K was first revealed to be responsible for more efficient replication of AIV in mammalian cells (MDCK) [19], and was subsequently found to be closely related to the enhanced pathogenicity in mice and the transmissibility in mammals in multiple AIV subtypes, including H1N1, H7N9, H5N1, H6N2, H4N6 and H9N2 [20, 33, 37, 41, 49–51]. The rapid development of deep sequencing technology has been utilized for studying adaptive mutations of viruses within hosts, and the PB2-E627K dynamic substitution process was confirmed in mammalian models and human infections by H10N8, H7N7, H5N1 and H7N9 AIV subtypes [20, 28–31].

In recent years, the PB2-E627V mutation has appeared with high frequency in several subtypes of AIV capable of infecting humans, drawing widespread attention. In 2015, an H7N9 AIV strain with the PB2-E627V substitution was isolated in Hunan, and revealed that this mutation increases the virus’s replication efficiency in mouse organs, enhancing its pathogenicity in mice. Additional research indicates that the E627V mutation in the PB2 protein can increase the virulence of H9N2 AIV in mice [32], and enhance the transmissibility of H7N9 AIV in ferrets [33]. Recently, three cases of human infection with the novel H3N8 AIV were reported in China, and one of the human-derived strains, A/Changsha/1000, carries the PB2-627 V molecular marker. Pathogenicity experiments demonstrated that it could kill mice [52]. Our previous studies have shown that the avian-derived H10N3 viruses harboring the PB2-E627V mutation are highly pathogenic in mice and can be transmitted between guinea pigs via respiratory droplets without prior adaptation [13, 23]. In this study, we found that the H10N3 strains carrying PB2-627 V and the mammalian signature PB2-627 K exhibit similar pathogenicity in mice, and are at least 100 times more pathogenic than strains carrying the avian signature PB2-627E (Figure 1). Furthermore, the H10N3 strains with the molecular marker PB2-627 V are capable of spreading among guinea pigs through direct contact and aerosol transmission, whereas strains carrying the PB2-627E do not possess the ability to transmit among guinea pigs. Typically, PB2-627 V is considered an intermediate state of the E to K mutation [20]. However, in this study, H10N3 strains carrying PB2-627 V demonstrate a capacity for full adaptation to mammalian hosts comparable to that of PB2-627 K. In addition, the PB2-E627V/K mutation does not impact the transmissibility of H10N3 in chickens, one key reason is that PB2-627E is non-essential for H10N3’s ability to spread in chickens, as other genomic sites can perform the same function too. This feature underscores the public health threat posed by H10N3 strains naturally carrying the PB2-627 V molecular marker, because poultry exposure is a major risk factor for human AIV infections. Furthermore, previous studies have demonstrated that PB2-627 V can be stably maintained in both avian and mammalian species [33].

As shown in Table 1, the PB2-E627K mutation is an important molecular marker for H5N1/N6 and H7N9 AIV subtype adaptation to humans, and among the H9N2 human-derived strains, the proportion of PB2-E627V is significantly higher than that of PB2-E627K. This suggests that H9N2 and other AIV subtypes of have different mechanisms of adaptation to mammals, so subsequent in-depth research into their molecular mechanism is required. In avian-derived H9N2 strains, the proportion of PB2-627 V is as high as 6.28%, indicating that this molecular marker can also persist in poultry. The internal genes of newly emergent AIV capable of infecting humans, such as H3N8, H7N9, H5N6, H10N8, and H10N3, are derived from avian H9N2 strains [7, 13, 53–55], which are prevalent in chickens in China [56]. Therefore, the widespread presence of the PB2-E627V mutation in avian-derived H9N2 is a significant factor contributing to the ability of these novel AIV to infect humans.

In summary, our findings demonstrate that the PB2-E627V mutation is key to the high pathogenicity of novel H10N3 in mice and its ability to be transmitted through the air in mammals. Additionally, the role of PB2-627 V in promoting AIV adaptation to mammals is comparable to that of PB2-627 K, and more importantly, PB2-627 V also appears to be equally suited to long-term persistence in poultry. Therefore, using PB2-627 V as a novel molecular marker to assess the epidemic potential of AIV is of great significance for preventing possible influenza pandemics in the future.

## Supplementary Information

**Additional file 1. Inflammatory cytokine of the novel H10N3 viruses with PB2-V/K/E mutation in mice.** Lung lavage samples from three mice in each virus-infected group (challenged with 10^5.0^ EID_50_) were collected for analysis of Inflammatory cytokine on 3 dpi.

## Data Availability

The data supporting the conclusions of this article are included within the article. Additional data used and/or analyzed during the current study are available from the corresponding author upon reasonable request.
