# Supplementary figures and images for: E627V mutation in PB2 protein promotes the mammalian adaptation of novel H10N3 avian influenza virus
Source: Vet Res. 2025 Jun 8;56:111. doi: 10.1186/s13567-025-01534-8 (PMC12145617; doi:10.1186/s13567-025-01534-8)

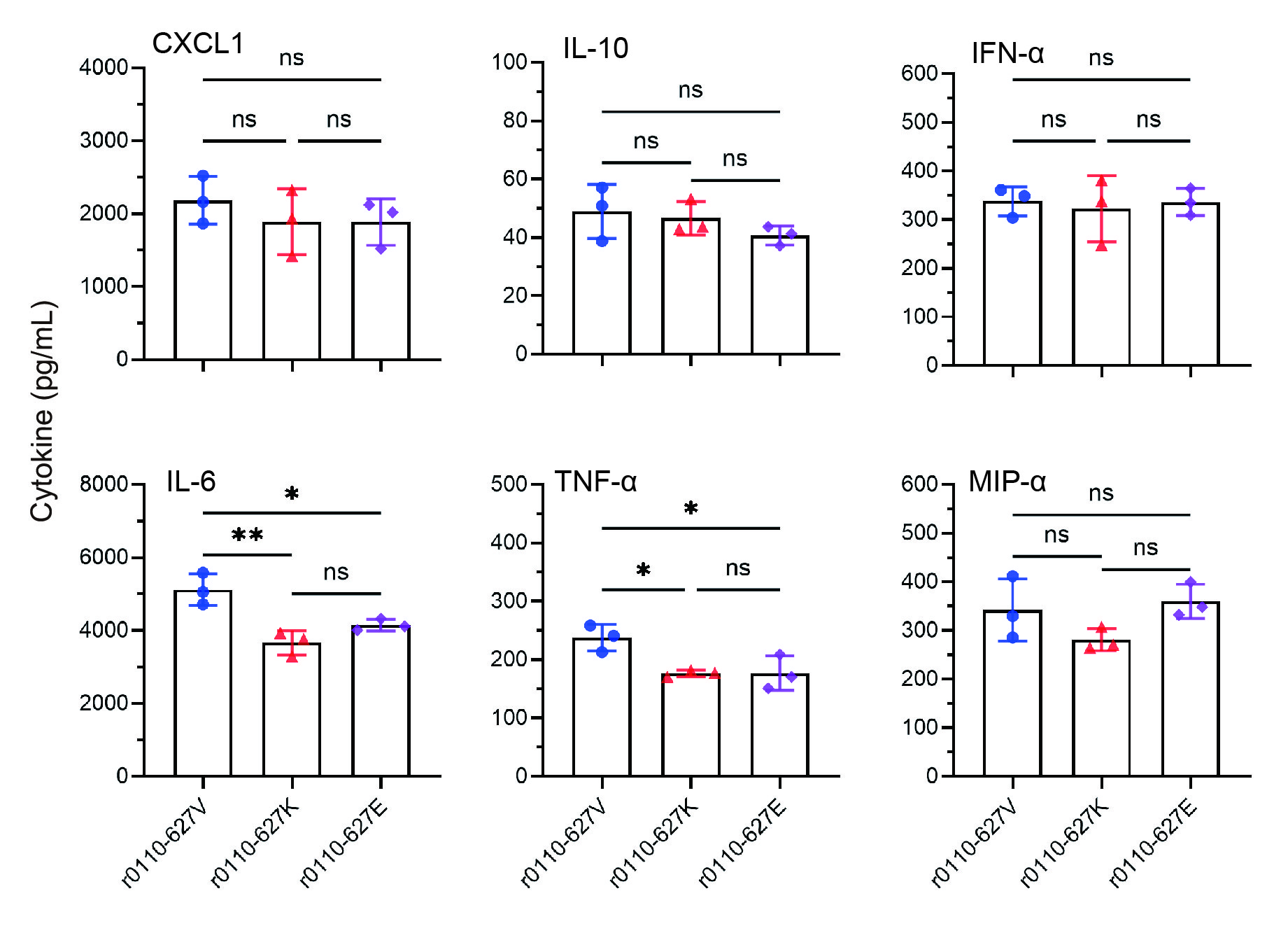

Supplement: Supplementary file 1 — Additional file 1. Inflammatory cytokine of the novel H10N3 viruses with PB2-V/K/E mutation in mice. Lung lavage samples from three mice in each virus-infected group (challenged with 105.0 EID50) were collected for analysis of Inflammatory cytokine on 3 dpi. [file 13567_2025_1534_MOESM1_ESM.doc]
